# Alterations in the expression of leukemia inhibitory factor following exercise: comparisons between wild-type and mdx muscles

**DOI:** 10.1371/currents.RRN1277

**Published:** 2011-11-22

**Authors:** Liam C Hunt, Chantal Anthea Coles, Christopher M Gorman, Elizabeth M Tudor, Gayle M Smythe, Jason D White

**Affiliations:** ^*^Faculty of Veterinary Science, University of Melbourne; ^†^Murdoch Childrens Research Insitute; ^¶^School of Community Health and Centre for Inland Health, Charles Sturt University and ^#^Murdoch Childrens Research Institute and School of Veterinary Science, University of Melbourne

## Abstract

Background: Leukemia inhibitory factor (LIF) is a pleiotropic cytokine, belonging to the interleukin-6 family of cytokines, that has been suggested to have positive effects on myogenesis following injury and to minimise dystrophic pathology in mdx mice. Previous reports have suggested that Lif mRNA is up-regulated in the limb and diaphragm muscles of mdx mice, in human cases of dystrophy and acutely following exercise. This study examined expression of Lif mRNA in the quadriceps muscles of mdx and wild-type mice that were either sedentary or allowed to exercise voluntarily for two weeks.

Results: Exercise caused a decrease in Lif mRNA expression in wild-type muscle, but this was not the case in mdx muscle. Lif mRNA levels in sedentary mdx mice were similar to those in exercised wild type muscles, and in mdx mice there was no further decrease in levels following exercise. Similar down-regulation of Lif mRNA was observed in the tibialis anterior and diaphragm muscles of mdx mice at three and six weeks of age respectively, compared with wild-type controls. Transcripts for the LIF receptor (Lifr) were also down-regulated in these mdx muscles, suggesting LIF activity may be minimised in dystrophic muscle. However fluorescent immunohistochemical labeling of LIF did not correlate with transcript expression data, as LIF immunoreactivity could not be detected in wild-type muscle, where mRNA expression was high, but was present in dystrophic muscle where mRNA expression was low. This study also described the translocation of membrane proteins, including LIFR, to the nuclei of syncytial muscle cells during differentiation and fusion. In addition this study demonstrates that survival of donor myoblasts injected into dystrophic muscle was enhanced by co-administration of recombinant LIF.

Conclusions: This study provides new evidence to support a role for LIF in normal muscle biology in response to exercise. Although expression levels of Lif transcript in mdx muscles were not consistent with previous studies, the detection of LIF protein in mdx muscle but not wild-type muscle supports a role for LIF in dystrophy. This study also provides evidence of the differential localisation of the LIFR, and the potential for anti-inflammatory actions of LIF that promote survival of transplanted myoblasts in dystrophic muscle.

*corresponding author: Jason White, Muscular Dystrophy Research Group, Murdoch Childrens Research Institute; email: jasondw@unimelb.edu.au

## Introduction

Leukemia inhibitory factor (LIF) is a pleiotropic cytokine belonging to the interleukin-6 (IL-6) family of cytokines, which all share the common receptor subunit gp130[1]. LIF was first named for being capable of inhibiting proliferation and promoting differentiation of the M1 murine myeloid leukemia cell line[2] [3], but as a pleiotropic cytokine has numerous effects on different cell types. LIF first became of interest in the context of skeletal muscle when it was shown to promote the *in vitro* expansion of myoblasts[4] [5], which has subsequently been shown to be caused not by enhancing proliferation but by increasing survival of myoblasts[6]. Since then LIF has been suggested to be important in regeneration of skeletal muscle because *Lif* mRNA is up-regulated following crush injury[7] [8], and knockout of LIF inhibits the formation of syncytial muscle cells (myogenesis) during crush-induced regeneration[9]. Conversely, addition of exogenous recombinant LIF can promote syncytial muscle cell growth and regeneration of the injured muscle[7] [9]. LIF is known to cause cellular effects on myoblasts by binding to a heterodimer of the gp130 and LIF receptor (LIFR) proteins[10] [11] [12] [13] which elicits downstream signaling via the common signaling mediators janus kinase (JAK), signal transducer and activator of transcription-3 (STAT3), phosphoinositide-3-kinase (PI3K) and mitogen activated protein kinase and extracellular-signal regulated kinase kinase (MEK)[14]. Signaling via these mediators can lead to inhibition of myoblast apoptosis[6] and inhibition of myogenic differentiation[15] [16] [17]. These cellular effects on myoblasts are believed to be responsible for the positive effects that LIF can have in regenerating muscle tissue.      

As well as being up-regulated in experimentally induced muscle injury and regeneration, *Lif* mRNA has been suggested to be up-regulated in cases of human muscle trauma and dystrophies[18], the dystrophic muscles of mdx mice[8], and acutely following a single three hour bout of concentric exercise in human vastus lateralis muscles[19]. Although the mdx mouse dystrophy is caused by similar genetic mechanisms to the human condition Duchenne Muscular dystrophy (DMD), causing a dystrophin protein deficiency, the murine phenotype is less severe than that in humans. However, while voluntary exercise by running on a wheel can induce a more severe pathology in mdx mice[20] [21] [22], the impact of exercise on expression and localisation of *Lif* and Lif receptor (*Lifr*) mRNA, and LIF and LIFR proteins, has not been investigated. Therefore, in the first part of this study expression of mRNA for *Lif*, as well as receptor components, in the quadriceps of mdx and wild-type mice performing voluntary wheel exercise was explored as was expression in the tibialis anterior and diaphragm of sedentary mdx mice at different ages. This was complimented with fluorescent immunohistochemical detection of LIF and LIFR proteins in normal and dystrophic muscle.

Exogenous LIF administration has been suggested to be beneficial not only for experimentally injured muscle and minimising dystrophic pathology in mdx mice by itself[23] [24] but also in conjunction with myoblast transplantation. Exogenous LIF, either presented in the myoblast injection solution or released into the dystrophic host muscle tissue via alginate gels, was shown to promote a higher level of dystrophin expression with myoblast transplantation into host mdx muscle compared to transplanted myoblasts alone[25] [26]. LIF does not induce proliferation of myoblasts and inhibits myogenic differentiation and fusion *in vitro* [5] [6] [17] and it is therefore unlikely that this would cause a greater number of donor myoblasts fusing to the regenerating host dystrophic myofibres thus increasing expression of dystrophin. One of the mechanisms by which LIF may enhance myoblast transplantation success is by protecting transplanted cells from the potentially harmful environment of the host muscle. Host CD4^+^ and CD8^+^ T lymphocytes and natural killer (NK) cells are believed largely responsible for the death of transplanted myoblasts[27] [28]. LIF promotes blastocyst immune tolerance in both pregnant mice and humans[29] [30] and is suggested to protect the blastocyst from immune rejection by up-regulation of human leukocyte antigen-G (HLA-G), a class I histocompatibility antigen which prevents maternal NK cell-mediated destruction of the blastocyst[31]. Therefore, the second part of this study aimed to determine if LIF could alter expression of histocompatibility antigens such as QA-2, the murine homolog of HLA-G, in myoblasts and promote survival of donor myoblasts injected into mdx muscles.

## Materials and Methods

### Mice

All experiments involving the use of mice were approved by and followed the University of Melbourne Animal Ethics Committee (AEC) guidelines. Approval was granted by the AEC under the animal ethics ID 0810901 and all procedures were conducted as described within the approved ethics submission. Mice were cared for in accordance with the ‘Australian Code of Practice for the Care of Animals for Scientific Purposes’ published by the National Health and Medical Research Council (Canberra, Australia). Mice were housed with 12 hour light/dark cycles and given access to food and water ad libitum.

### Exercised C57BL10/ScSn and C57BL10/mdx 

A total of 12 C57BL10/ScSn and 12 C57BL10/mdx mice, all male and aged 10 weeks at the onset of experimentation, were used for the exercise study. For each mouse strain, 6 mice were sedentary controls and 6 mice had access to a running wheel. Exercised animals were kept individually in custom built cages containing exercise wheels with sensors to monitor the movement, and allowed to run at their own free will for two weeks. Sedentary mice were kept in standard mouse cages. After the exercise period, mice were sacrificed by carbon dioxide asphyxiation and the entire quadriceps muscle group from the right side was removed and frozen in liquid nitrogen for RNA extraction and the quadriceps muscles from the left side were mounted and frozen in liquid nitrogen quenched isopentane for histology. Logs of the distance run and speeds reached were recorded as well as time since the last bout of exercise to sampling.

### Sedentary C57BL10/ScSn and C57BL10/mdx

For examining expression of Lif and receptor transcripts by quantitative real-time PCR (qPCR) four male and four female mice of each of the C57BL10/mdx and C57BL10/ScSn strains at 2, 3, 6, 12 and 24 weeks of age were sacrificed by carbon dioxide asphyxiation. The diaphragm and tibialis anterior muscles were excised and frozen for RNA extraction in order to perform qPCR. The contralateral tibialis anterior was also excised and mounted in 5% (w/v) tragacanth which was then frozen in liquid nitrogen cooled isopentane for histological use. In order to perform histochemical and immunohistochemical staining the frozen tibialis anterior muscles were cut into 10μm sections on a Leica CM3050S cryostat and mounted on Starfrost microscope slides (Knittel Glaser, Bielefeld, Germany) and kept frozen for future use.

### RNA extraction, reverse transcription and qPCR

Total RNA was extracted from cultures grown in 6-well plates using SV Total RNA Isolation System (Promega, Madison, USA). RNA was extracted from muscle tissue by homogenising the tissue in 1mL of Tri-reagent (Molecular Research Centre, Cincinatti, USA). To this 200μL of chloroform was added and mixed thoroughly followed by centrifugation at 14,000g for 15 minutes at 4^o^C. The aqueous layer was removed and added in equal volume to a solution comprising one part 90% ethanol and one part SV lysis solution from SV Total RNA Isolation System. The resulting solution was then processed through SV Total RNA Isolation System columns and the standard procedure followed. The RNA was eluted from the columns in 50µL of nuclease free water and the concentration and purity determined by spectrophotometric methods. RNA samples were added to a reverse transcription reaction at 1ug of total RNA reverse transcribed with MML-V Reverse Transcriptase (Promega, Madison, USA) at 42^o^C for one hour. The resulting complementary DNA (cDNA) was then used for qPCR.      

Each qPCR reaction contained 0.5µL cDNA, 5µL ‘GoTaq qPCR master mix’ (Promega, Madison, USA), 4µL nuclease free water and 0.25µL of both forward and reverse oligonucleotide primers at 20µM. qPCR was performed on a LightCycler 480 (Roche Applied Science, Basel, Switzerland) and thermally regulated with the following parameters:- Pre-incubation at 50^o^C for five minutes followed by 95^o^C for five minutes and then 40 cycles of 95^o^C for 20 seconds for denaturing, 60^o^C for 30 seconds of annealing and 72^o^C for 30 seconds of amplicon extension. The LightCycler 480 program was used to derive cycle threshold (Ct).

Oligonucleotide primers were designed for specific transcripts using ‘Primer-BLAST’ available on the website http://www.ncbi.nlm.nih.gov, spanning introns, where possible, to further reduce chances of detecting genomic DNA within samples and to produce amplicons of 50-250 base pairs (bp) for detection by ‘Sybr Green’ chemistry based qPCR. Primers were obtained through Sigma-Aldrich’s custom oligonucleotide service. Primers were tested for efficiency (E) by serial dilution of cDNAs. Primers were only accepted for use if 1.8≤E≤2.2. Oligonucleotide sequences are presented in along with the PubMed Gene ID number for identification in Table 1. qPCR Data was expressed as the mean of normalised expression in order to display linear levels of transcripts of interest normalised to the housekeeper hypoxanthine-guanine phosphoribosyltransferase (Hprt), which was the most adequate housekeeper as it possessed the least variability across all samples compared to other housekeepers genes tested including Ppia, Gapdh and Actb using the “Bestkeeper” excel based tool[32]. For determining normalised expression the following formula was used:-

 Normalised expression = (A^x^)/(B^y^).  

Where A is the efficiency of amplification of the housekeeper, B is the efficiency of the gene of interest, x is the housekeeper Ct and y is the gene of interest Ct.

**Table:** 

Gene name	Protein name	ID	Forward (5’-3’)	Reverse (5’-3’)
*Hprt*	HPRT	15452	GATTAGCGATGATGAACCAGGTT	TCCAAATCCTCGGCATAATGAT
*Emr1*	F4/80	13733	ACAGCCACGGGGCTATGGGA	GCACCCAGGAGCAGCCCCAG
*Il6st*	gp130	16195	CATAGTCGTGCCTGTGTGCT	GTGACCACTGGGCAATATGA
*Lif*	LIF	16878	CAAGAATCAACTGGCACAGC	AGTGGGGTTCAGGACCTTCT
*Lifr*	LIFR	16880	AGAAGAACTGGCTCCCATTG	GGATGTCGTCCCATTTCACT
*Myod1*	myoD	17927	TACAGTGGCGACTCAGATGC	TAGTAGGCGGTGTCGTAGCC
*Myog*	myogenin	17928	AGTGAATGCAACTCCCACAG	ACGATGGACGTAAGGGAGTG
*Qa2*	QA-2	110558/15018	AGCAGGCTGGTATTGCAGAG	AACTGCCAAGTCAGGGTGAT

#### **Table 1 Oligonucleotide primer sequences used for all reverse transcription qPCR**

### Notexin induced regeneration of the tibialis anterior

Notexin induced regeneration was utilised in order to assess LIFR immunoreactivity in the tibialis anterior of regenerating muscle. Male 10 week old C57BL6 mice were anaesthetised and received a single injection of 50µL of 10µg/mL notexin (Latoxan, Valence, France) made up in saline longitudinally into both tibialis anterior muscles with a 29G 1mL BD SafetyGlide syringe (BD, Franklin Lakes, USA). The mice were allowed to recover and at different days after notexin injection asphyxiated by carbon dioxide and the tibialis anterior muscles dissected. Tibialis anterior muscles were frozen for histology by mounting in 5% (w/v) tragacanth which was then frozen in liquid nitrogen cooled isopentane. In order to perform immunohistochemical staining the frozen tibialis anterior muscles were cut into 10μm sections and mounted on glass microscope slides and kept frozen for future use.

### Histochemical and immunofluorescent staining    

In order to assess histology of muscles haemotoxylin and eosin staining was performed on the frozen sections mounted on glass slides by immersing the slides first in 70% ethanol followed by distilled water and then left in Harris haemotoxylin single strength (ProSciTech, Kirwan, Australia) for 30 seconds. Slides were then immersed in distilled water followed by Scott’s tap water (distilled water with eight drops of ammonia per 300mL of water) and 70% ethanol before being immersed in a solution of 1% (w/v) Eosin-Y (ProSciTech, Kirwan, Australia) in 70% ethanol for one minute. Sections were then dehydrated through two steps each of 95% and 100% ethanol, cleared in xylene and finally mounted with Entellan (ProSciTech, Kirwan, Australia) to coverslips.

Immunofluorescent staining was conducted on unfixed frozen tissue sections and C2C12 myoblast cells fixed with 100% methanol for a range of proteins. Where co-staining of multiple proteins occurred on the same section, all primary antibodies were combined in the same incubation solution, and a different combination of fluorophores conjugated to secondary antibodies was utilised as required. Non-specific staining was blocked with 5% (v/v) donkey serum and 2% (w/v) bovine serum albumin (BSA) in PBS for one hour at room temperature. Sections were incubated overnight at 4ºC, with primary antibodies against rabbit anti-LIFR (Santa Cruz Biotechnology, Santa Cruz, USA), goat anti-LIF (Santa Cruz Biotechnology), mouse anti-desmin (Sigma-Aldrich, Castle Hill, Australia), rat anti-laminin a2 (Santa Cruz Biotechnology), or mouse anti- myosin heavy chain (skeletal fast) (Sigma-Aldrich), all diluted at 1:200 in PBS.

Sections were then washed and incubated with fluorescent secondary antibodies (all from Invitrogen, Carlsbad, USA) as required to bind the primary antibodies (donkey anti-rabbit AlexaFluor-488, chicken anti-goat AlexaFluor-594, donkey anti-mouse AlexaFluor-594, or donkey anti-rat AlexaFluor-594) diluted at 1:250 in PBS for 45 minutes in the dark and counterstained with 10µg/mL 4’,6-diamidino-2-phenylindole (DAPI) in PBS for 1 minute. Sections were mounted with glass coverslips and visualised by fluorescence microscopy. Species-specific IgG negative controls were performed on representative sections for all primary antibodies to ensure that immunoreactivity was not the result of non-specific binding of IgG. Where the primary antibody was raised in a mouse, blocking of endogenous mouse IgG was performed using a mouse on mouse fluorescein kit (Vector Laboratories, Burlingame, USA). Where neuromuscular junction (acetylcholine receptors) labeling was also required, this was achieved by incubating sections with an AlexaFluor-488 conjugated α-bungarotoxin (Invitrogen, Carlsbad, USA), diluted 1:1000 in PBS for 20 minutes in the dark prior to the DAPI labeling step.

### Primary and C2C12 myoblast cultures

Skeletal muscle myoblasts were extracted from muscle tissue isolated from the hind limbs of C57BL10/ScSn mice. Four week old male C57BL10/ScSn mice were euthanised by carbon dioxide inhalation and the hind limb muscles dissected. Obvious non-muscle tissue such as fat, nerve and tendon tissue was removed and discarded. Muscle tissue was finely minced using scissors and transferred to a conical mixing flask containing 10mL per gram of tissue of 1.5U/mL type I collagenase obtained from Worthington Biochemicals (Vassar, USA) and 2.4U/mL dispase from Invitrogen (Carlsbad, USA) and digested with stirring at 37^o^C for 45 minutes. Digested cells were filtered through a 70µm filter and pelleted at 2,000g for 5 minutes. Pelleted cells were re-suspended in HAMS F10 (Invitrogen, Carlsbad, USA) containing 20% (v/v) foetal bovine serum (FBS), 2.5ng/mL FGF-2 (Invitrogen, Carlsbad, USA), 100U/mL penicillin and 100µg/mL streptomycin and “pre-plated” by incubating in an uncoated tissue culture flask at 37^o^C for 45 minutes to allow fibroblasts and other more adhesive cells to attach while retaining myoblasts primarily in the supernatant. The supernatant was then plated into flasks that had been previously coated with gelatin by incubation at 37^o^C with 1% (w/v) gelatin for one hour. The culture was then expanded to enrich for myoblasts by re-iterative pre-plating and to obtain the number of myoblasts required for experimentation[33].

Experiments involving addition of recombinant LIF to myoblasts was performed by culturing both primary and C2C12 myoblasts in 6 well plates and adding 10ng/mL of recombinant murine LIF (Millipore, Billerica, USA) to the growth media of DMEM containing 10% (v/v) FBS, 100U/mL penicillin and 100µg/mL streptomycin for 24 hours. Cultures were then washed and lysed for RNA extraction.

### Biotinylation of myoblast cell surface proteins

Proteins on the surface of C2C12 myoblast cells were biotinylated with Sulfo-NHS Biotin (Thermo Scientific, Scoresby, Australia) and detected with streptavidin conjugated AlexaFluor-594 (Invitrogen, Carlsbad, USA). C2C12 cells were trypsinised into suspension, washed with ice cold PBS (pH 8.0) and re-suspended at a concentration of 1x106 cells/mL in PBS (pH 8.0). Biotin reagent was added at a final concentration of 2mM to the cell suspension and incubated on ice for 30 minutes. Cells were then washed with PBS and remaining Sulfo-NHS Biotin was quenched with 100mM glycine in PBS. Following biotinylation cells were seeded onto plates and cultured. For detection of biotinylated proteins either differentiated whole cultures or pelleted intact nuclei were fixed with 100% methanol at 4^o^C for 10 minutes washed with PBS and blocked with 5% (w/v) BSA in PBS for one hour at room temperature. Samples were then incubated with either 1:200 dilution of LIFR (C-19) rabbit polyclonal antibody (Santa Cruz Biotechnology, Santa Cruz, USA) overnight at 4^o^C and 8µg/mL AlexaFluor-488 donkey anti-rabbit IgG (Invitrogen, Carlsbad, USA) along with 1µg/mL streptavidin conjugated AlexaFluor-594 or just streptavidin conjugated AlexaFluor-594 for 45 minutes in the dark. Samples were counterstained with 10µg/mL 4’,6-diamidino-2-phenylindole (DAPI) in PBS for 1 minute. Samples were then visualised by fluorescence microscopy.

### Nuclear isolation and Western immunoblotting

To separate intact nuclei from C2C12 cultures, in order to visualise proteins contained within the nuclei, for immunocytochemistry and lysed nuclei to detect nuclear proteins by Western immunoblotting, a differential lysis procedure was utilised. The cytoplasm of cells was lysed by incubation with 50 mM Tris-HCl (pH 7.5), 0.5% Triton X-100, 137.5 mM sodium chloride, 10% Glycerol, 5 mM EDTA and cOmplete protease inhibitor cocktail for 15 minutes on ice followed by pelleting the intact nuclei by centrifugation at 5,000g for 15 minutes at 4^o^C. The pellet was washed once with the lysis buffer and re-pelleted. All supernatant was kept as the cytoplasmic protein fraction and incubated at 95^o^C for 5 minutes with an equal volume of a reducing gel loading buffer containing 100mM Tris pH 6.8, 2% (w/v) SDS, 5% (v/v) ß- mercaptoethanol, 15% (v/v) glycerol and 0.1% (w/v) bromophenol blue. The intact nuclei were then lysed by incubation at 95^o^C for 5 minutes with reducing gel loading buffer to the same total volume as the cytoplasmic fraction and DNA was sheared by passing the solution through a 29G needle. Nuclear protein fractions were found to be greatly enriched for nuclear proteins compared to cytoplasmic protein fractions by abundance of Histone H3 determined by Western immunoblotting described below.

Protein expression analysis of cytoplasmic and nuclear fractions was conducted by resolving an equal total protein aliquot of each sample by standard SDS-PAGE, transferring proteins to a PVDF transfer membrane (Pall Corporation, Melbourne, Australia), blocking with 5% (w/v) skim milk in PBS with 0.1% Tween-20 overnight at 4^o^C, followed by primary antibodies, either anti-LIFR or anti-histone H3 (D1H2) XP Rabbit mAb (Cell Signaling Technologies, Danvers, USA) diluted in blocking solution overnight at 4^o^C. Horseradish peroxidase conjugated antibodies were used to detect the primary antibodies by incubation for one hour at room temperature, and expression analysed by standard enhanced chemiluminescence.

### Myoblast transplantation and survival      

Cultured male C57BL10/ScSn myoblasts were trypsinised, pelleted and then resuspended at a concentration of 200,000 cells per 10μL in either PBS or PBS containing recombinant LIF. Six week old female mdx mice were anaesthetised and received a single injection of the 10μL solution containing 200,000 male myoblasts either with or without LIF into the tibialis anterior with a 10µL Hamilton gastight syringe #1801 (Hamilton, Reno, USA). Six tibialis anterior samples were collected per group either immediately after myoblast injection or from one to three days after the injection with or without LIF and snap frozen for genomic DNA extraction in order to quantitate the persisting Y chromosomes, from the donor cells, in the muscle. Solutions of 10μL containing 200,000 donor myoblasts were also used for DNA extraction to compare the intended number of myoblasts injected to what was actually received in the muscle immediately after injection. 

Genomic DNA from the frozen muscle samples and cells was extracted with Wizard Genomic DNA purification kit (Promega, Madison, USA). The concentration of the eluted DNA was determined by spectrophotometric methods and samples were diluted in nuclease free water to give 20ng/μL for use in qPCR for detection of *Zfy*, a Y chromosome marker. The diluted samples were added to qPCR reaction mixtures as described above using primers for *Zfy[28]*, which were: forward 5’-3’- TGG AGA GCC ACA AGC TAA CCA and reverse 5’-3’- TCC CAG CAT GAG AAA GAT TCT TC. The qPCR was performed and Ct values used to determine the relative level of *Zfy* present per ng of DNA extracted from the muscles.

### Statistical analysis

Where data showed normal distribution, Graphpad Prism (Graphpad Software Inc., La Jolla, USA) was used to perform statistical tests of significance. Student’s t-test was used to compare the mean and error of data where only two groups were analysed. Analyses of multiple groups were performed using one-way analysis of variance (ANOVA), with Dunnet’s or Bonferroni post-hoc test when all treatments were compared to a single control or all treatments compared to each other respectively. For analysis of Ct values obtained from qPCR, which is not linearly proportional to a change in transcript concentration and where normal distribution is not expected, a pair-wise fixed reallocation randomisation test relative expression software tool (REST) was used to determine significance[34]. A 95% confidence interval was accepted where P≤0.05 was deemed significant. All data is graphically represented as the mean ± the standard error of the mean (SEM) and sample size (n) is reported for all experiments. 

## Results

### Expression of *Lif, Lifr and Il6st* mRNA in the quadriceps of exercised and sedentary mdx and wild-type mice

Histology of exercised mdx quadriceps muscles indicated that a greater degree of damage was achieved with voluntary exercise as numerous necrotic lesions with mononucleated cells and small myotubes were present compared to sedentary mdx (Figure 1). The histology also suggested that small amounts of damage may occur in wild-type mice with this voluntary exercise regime as there was a very small degree of central nucleation present and increased numbers of nuclei around the periphery of myofibres compared to sedentary.

**Figure:**
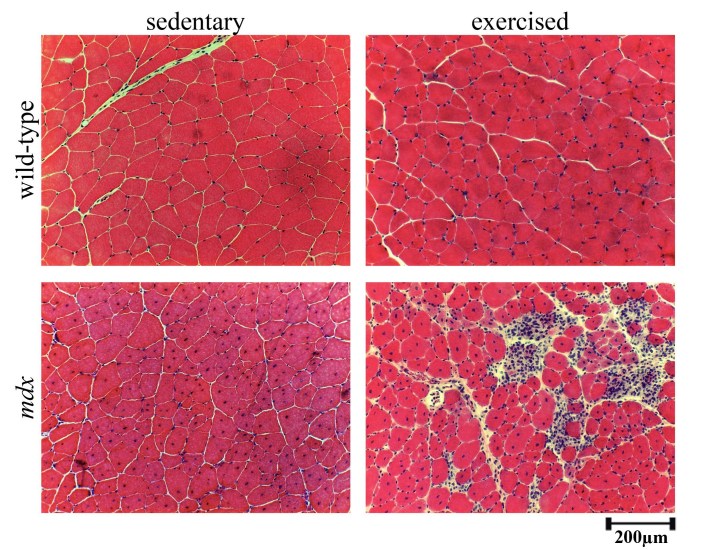
***Figure 1 Histology of wild-type and mdx exercised and sedentary quadriceps muscles***

*Representative images of haemotoxylin and eosin stained sections from the quadriceps of wild-type and mdx mice with exercise or without (sedentary). Scale bar represent 200μm.*

*Lif*, *Lifr *and *Il6st* mRNA expression was analysed in quadriceps muscles from sedentary and exercised wild-type (C57BL10/ScSn) and mdx mice. In sedentary mdx mice, *Lif* and *Lifr* mRNA were significantly down-regulated compared with sedentary wild-type controls (Figure 2A-B). There were no strain-specific differences in *Il6st* mRNA expression in the quadriceps muscles from sedentary mice (Figure 2C). Exercise caused a significant decrease in *Lif* mRNA expression in the quadriceps muscles of wild-type, but not mdx, mice (Figure 2A). Exercise did not affect expression of *Lifr* or *Il6st* mRNA in either mouse strain (Figure 2B, 2C).

**Figure:**
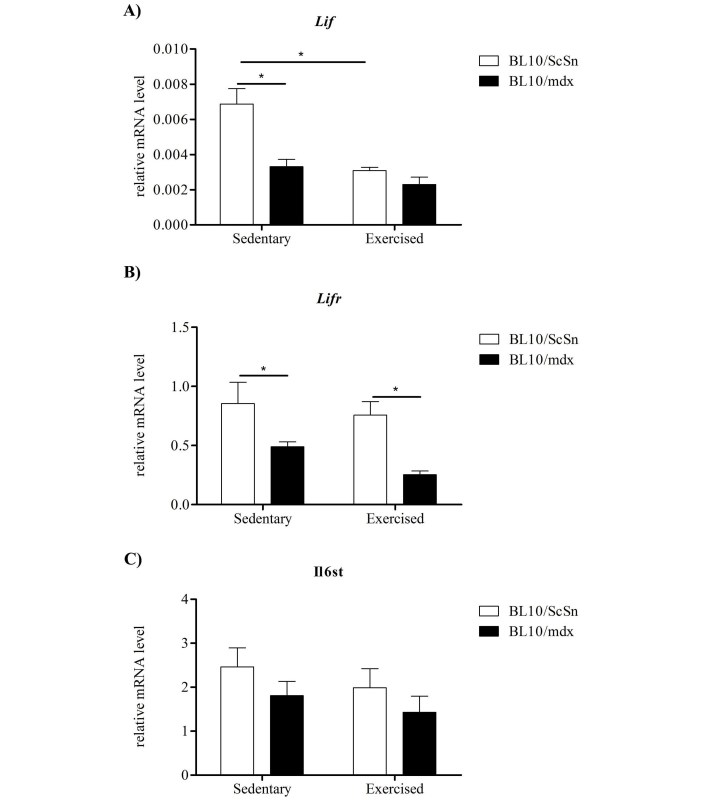
***Figure 2 Expression of Lif, Lifr and Il6st transcripts in exercised and sedentary dystrophic and wild-type quadriceps muscles***

Relative mRNA levels of A) Lif, B) Lifr and C) Il6st determined by qPCR in the quadriceps of C57BL10/mdx dystrophic and C57BL10/ScSn wild-type muscle after 2 weeks of wheel running exercise or sedentation. Data was analysed by the pair-wise fixed reallocation randomisation test REST comparing across genotype and exercise versus sedentary, * represents P≤0.05 (n=6)

Both mdx and wild-type mice were active and ran similar distances with the majority of running performed during the night. There was however a slight bias towards increased activity in the wild-type mice. The time at which the muscles were sampled after the last bout of exercise was recorded, as *Lif* mRNA has been shown to be up-regulated immediately after a single bout of exercise in humans compared to unexercised muscle[19]. The average time between the last run and sampling was approximately 4.5 hours. There were no obvious relationships between mRNA levels and the time lapse between the last run and tissue sampling (results not shown).

### Expression of *Lif, Lifr and Il6st* mRNA in tibialis anterior and diaphragm of sedentary mdx compared to wild-type mice at different ages

The observed decrease in expression of *Lif* and *Lifr* mRNA in sedentary mdx quadriceps muscles compared with wild-type controls was discordant with previous studies that reported up-regulation of *Lif* mRNA in the mdx limb and diaphragm muscles[8]. Therefore this was examined more thoroughly across several ages in the tibialis anterior limb and diaphragm muscles of sedentary mdx and age-matched wild-type mice.

Markers of myogenesis and inflammation were first examined to provide a timeline of myogenic events in the wild-type and mdx muscles. Transcript levels of *Myod1 *and* Myog*, the genes which encode the myogenic regulatory proteins MyoD and myogenin respectively, were significantly increased in the mdx tibialis anterior and diaphragm muscles compared to wild-type controls from the ages 6 weeks and beyond (Figure 3) indicating that the process of myogenic differentiation and formation of central nucleated regenerated fibres had occurred coinciding with the presence of central nucleated fibres in the histology. Transcripts for the gene *Emr1,* encoding the macrophage protein F4/80, were also analysed as a marker of the extent of inflammation[35]. Transcripts for *Emr1* were significantly increased in mdx tibialis anterior and diaphragm muscles compared to wild-type at only 6 and 12 weeks of age (Figure 3), suggesting a higher proportion of macrophages present in the muscle at these ages. This is in accordance with previous reports that show increased expression of myogenic regulatory factors and inflammatory markers[36] [37] and provides an internal positive control indicating that the methodology was accurate.

**Figure:**
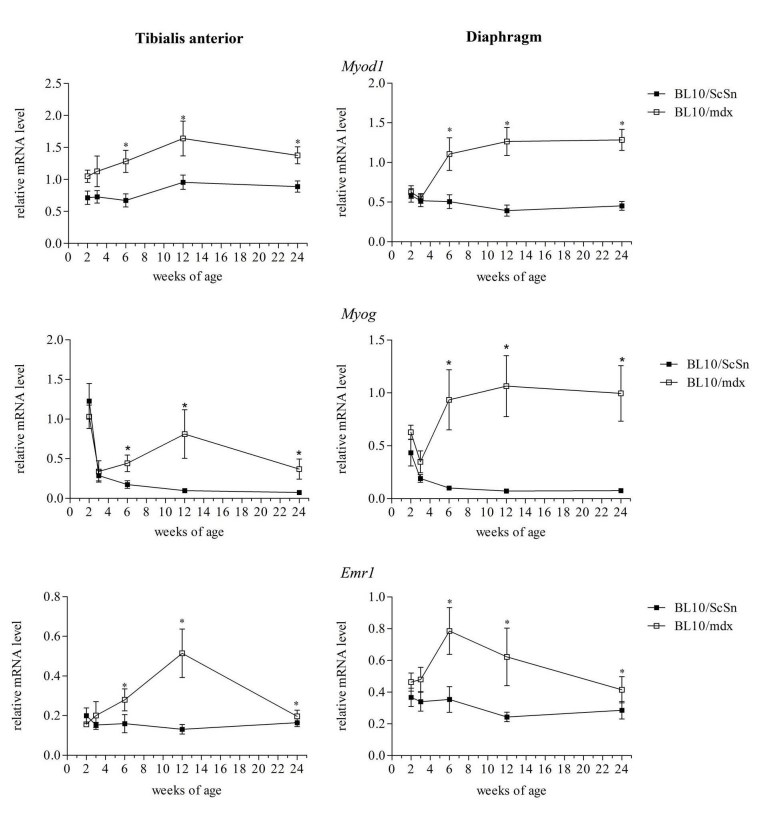
***Figure 3 Transcriptional changes of Myod1, Myog and Emr1 in mdx and wild-type muscle with age***

Relative mRNA levels of Myod1, Myog and Emr1 in tibialis anterior and diaphragm muscles of mdx and wild-type mice from 2-24 weeks of age. Transcript levels from 4 male and 4 female mice from each group were consolidated. Data was analysed by the pair-wise fixed reallocation randomisation test REST comparing mdx to wild-type, * represents P≤0.05 (n=8)

*Lif*, *Lifr *and *Il6st* mRNA expression was examined from 4 male and 4 female mdx and wild-type mice (Figure 4). Gender specific differences in *Lif*, *Lifr* and *Il6st* expression were not apparent (results not shown) and so data from male and female were combined. *Lif* mRNA showed a significant peak in expression at 3 and 6 weeks of age in the tibialis anterior and diaphragm muscles respectively from wild-type mice, and this peak was not observed in mdx muscles in which *Lif* mRNA levels remained relatively consistent at all ages (Figure 4). This suggested that *Lif* mRNA is normally up-regulated in muscle during growth and development however did not occur in the mdx muscles. Similarly *Lifr* mRNA was significantly higher in the wild-type muscles than mdx at 12 weeks of age in the tibialis anterior and 6 and 12 weeks in the diaphragm (Figure 4). Relative mRNA levels of *Il6st*, which encodes the receptor protein gp130, were not significantly altered in either muscle or at any age compared to wild-type (Figure 4).

**Figure:**
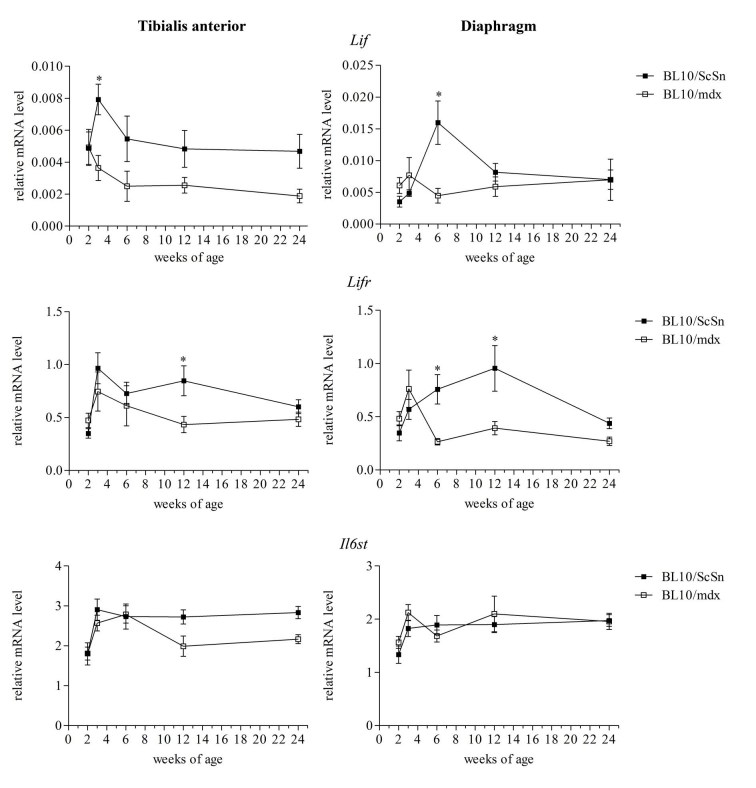
***Figure 4 Transcriptional changes of Lif, Lifr and Il6st in mdx and wild-type muscle with age***

Relative mRNA levels of Lif, Lifr and Il6st in tibialis anterior and diaphragm muscles of mdx and wild-type mice from 2-24 weeks of age. Data from combined male and female samples were analysed by the pair-wise fixed reallocation randomisation test REST comparing mdx to wild-type, * represents P≤0.05 (n=8)

### LIF and LIFR protein expression in normal and dystrophic muscle

Although there was a trend towards increased *Lif* mRNA in wild-type muscle compared to mdx there was little LIF protein immunoreactivity in the tibialis anterior from 6 week old wild-type mice (Figure 5A). LIFR immunoreactivity however was evident in 6 week old wild-type muscles where it was present sporadically at the myofibre periphery. When compared to laminin immunoreactivity, which localises to the endomysium surrounding myofibres, LIFR immunoreactivity appeared to localise to nuclei associated with the endomysium of wild-type muscles, suggesting either satellite cells or peripheral myofibre nuclei (Figure 5B). LIFR immunoreactivity was also present near the endomysium where nuclei were not present suggesting possible localisation to myofibre sarcolemma. Sporadic LIFR immunoreactivity around myofibres was reminiscent of acetylcholine receptor localisation in motor endplates of myofibres and therefore was compared to fluorescent α-bungarotoxin labeling of acetylcholine receptors (Figure 5C). LIFR immunoreactivity appeared particularly intense in regions where motor end plates were present however was not exclusive to the motor endplates. 

Expression of LIF and LIFR was also examined in the mdx tibialis anterior muscles at different ages. At 2 weeks of age in the mdx tibialis anterior both LIF and LIFR immunoreactivity appeared similar to that observed in 6 week old wild-type muscle with sporadic LIFR immunoreactivity surrounding fibres and no detectable LIF immunoreactivity (Figure 5D). By 6 weeks of age though when centrally nucleated fibres were present in the dystrophic muscle, LIFR immunoreactivity coincided with the central nuclei and LIF immunoreactivity was present on the numerous mononucleated cells surrounding regenerated fibres (Figure 5E). At 12 weeks of age LIF immunoreactivity was less apparent although large numbers of mononucleated cells were still present in lesions, whilst LIFR immunoreactivity was particularly intense in the lesions containing numerous mononucleated cells (Figure 5F). It was curious that LIFR immunoreactivity appeared to localise to the central nuclei of regenerated myofibres as LIFR is typically described as a cell surface receptor responding to extracellular ligands such as LIF secreted from cells. This was further examined in other models where syncytial muscle cells are formed both *in vitro* and *in vivo*.

**Figure:**
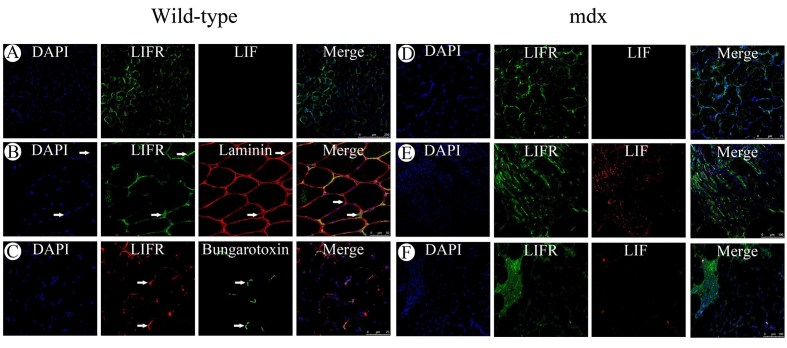
***Figure 5 Immunofluorescent detection of LIF and LIFR in normal and dystrophic muscle***

*Immunofluorescent detection of LIF and LIFR in histology sections from the tibialis anterior muscles of 6 week old C57BL10/ScSn wild-type mice (left). A) Dual labeling of LIFR (green) and LIF (red). B) Dual labeling of LIFR (green) and laminin (red) with arrows indicating LIFR immunoreactivity that coincides with the sarcolemma (top) or a nuclei in close association with the endomysium (bottom). C) Dual labeling for LIFR (red) and acetylcholine receptors by fluorescently conjugated α-bungarotoxin (green) with arrows indicating intense LIFR immunoreactivity coinciding with bungarotoxin fluorescence. Immunofluorescent detection of LIF and LIFR in histology sections from the tibialis anterior muscles of C57BL10/mdx mice (right). D) Dual labeling of LIFR (green) and LIF (red) in tibialis anterior from 2 week old mdx mice. E) Dual labeling of LIFR (green) and LIF (red) in tibialis anterior from 6 week old mdx mice. F) Dual labeling for LIFR (green) and LIF (red) in tibialis anterior from 12 week old mdx mice.*

### LIFR immunoreactivity in the nuclei of syncytial muscle cells

LIFR immunoreactivity was observed by fluorescent confocal microscopy in the central nuclei of regenerated fibres in the tibialis anterior from 12 week old mdx mice (Figure 6). LIFR immunoreactivity was most apparent in mdx fibres that showed more intense desmin immunoreactivity, suggesting less mature myofibres or myotubes, whilst larger and more mature appearing fibres displayed less obvious LIFR immunoreactivity within the central nuclei (Figure 6). This was also apparent in syncytial muscle cells formed after notexin injury and regeneration of the tibialis anterior (Figure 6). By 5 days after notexin injection the first myotubes were present; these showed intense desmin immunoreactivity throughout the cytoplasm as well as LIFR immunoreactivity that appeared exclusive to the central nuclei of these myotubes (Figure 6). When these myotubes or myofibres were more mature and desmin immunoreactivity was less intense, by 14 days after notexin injection, LIFR immunoreactivity remained in the central nuclei however was also observed surrounding the edges of these syncytial muscle cells (Figure 6). *In vitro* C2C12 myoblasts that were proliferating showed cell surface LIFR immunoreactivity that would be expected, however when differentiation was induced and the myoblasts fused to form myotubes, LIFR immunoreactivity was evident within the nuclei of the syncytial myotubes (Figure 6). Taken together, these data suggest that LIFR is present on the myoblast membrane and translocates to the nucleus upon differentiation and fusion, with a subsequent reversion to membrane localisation on myofibre maturation.

**Figure:**
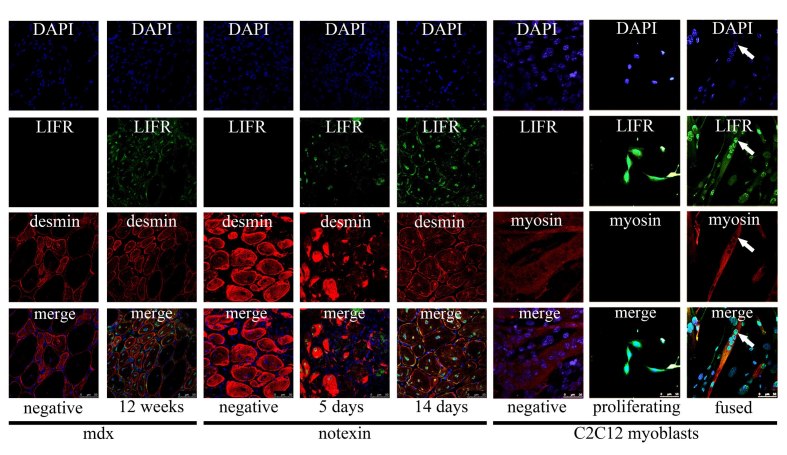
***Figure 6 Immunofluorescent detection of LIFR in the nuclei of syncytial muscle cells***

*Immunofluorescent labeling of LIFR (green) combined with either desmin (red) for in vivo syncytial muscle cells in mdx tibialis anterior (left), notexin injected tibialis anterior (middle) and myosin heavy chain (red) for in vitro syncytial muscle cells in C2C12 cultures (right). Negative indicates LIFR antibody negative controls where the LIFR antibody was pre-adsorbed with the antigen that the antibody was raised against; no non-specific immunoreactivity was observed with LIFR antibody negative controls. Positive labeling is from 12 week old mdx (left), 5 and 14 days after notexin injection (middle) and proliferating C2C12 myoblasts and fused C2C12 myotubes 48 hours after induction of differentiation (right).*

The possibility of translocation of myoblast cell surface proteins and LIFR to myotube nuclei was investigated by biotinylation of cell surface C2C12 myoblast proteins followed by differentiation. Proliferating C2C12 myoblasts that had cell surface proteins biotinylated were induced to differentiate and the localisation of biotinylated proteins examined with fluorescently conjugated streptavidin. Both negative controls lacking either biotinylation or the conjugated streptavidin displayed no fluorescent labeling (Figure 7A (a) & (b)). Biotinylation of proliferating myoblasts showed fluorescent labeling on the plasma membrane of only some myoblasts (Figure 7A (c)). These biotinylated proteins appeared to translocate as some myotube nuclei showed fluorescent labeling (Figure 7A (d)) indicating the presence of biotinylated proteins that were previously located on the plasma membrane. This provided proof of principle that cell surface proteins on myoblasts may translocate to nuclei upon differentiation and fusion into syncytial cells. Intact nuclei were also isolated from these myotube cultures to ensure that the immunoreactivity could be detected in the nuclei alone. LIFR immunoreactivity co-localised with immunofluorescent labeling of biotinylated proteins with streptavidin within the isolated nuclei (Figure 7B) suggesting it could potentially be one of the proteins translocating into myotube nuclei. Cytoplasmic and nuclear enriched lysates were extracted from myotube cultures and western immunoblotting of LIFR performed on the lysates to determine if native 190kDa LIFR was present. The cytoplasmic fractions displayed immunoreactive bands at 190kDa suggesting that full-length LIFR may have been present either in the cytoplasm or plasma membrane of the myotubes which composed the cytoplasmic lysate (Figure 7C lanes 1&2). Enriched nuclear fractions showed several immunoreactive bands however a 190kDa band was not present (Figure 7C lanes 3&4) suggesting that if the LIFR protein were responsible for the immunoreactivity observed within myotube nuclei it was not likely native LIFR. Histone H3 immunoblotting indicated that the nuclear fractions were indeed greatly enriched for nuclear proteins compared to the cytoplasmic fractions (Figure 7D).

**Figure:**
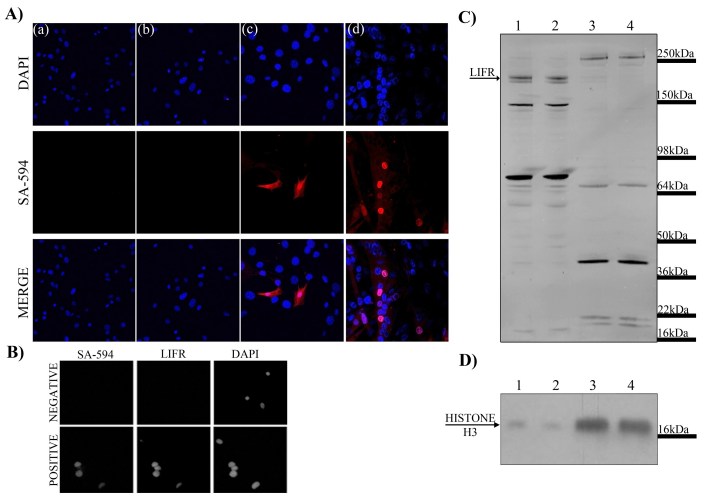
***Figure 7 Examination of nuclear translocation of cell surface proteins during myoblast differentiation***

*A) Immunofluorescent detection within C2C12 myoblast and myotube cultures by DAPI for nuclei (blue) and streptavidin-594 (SA-594; red) to visualise biotinylated proteins. Panel (a) represents the negative control lacking the biotinylation procedure; (b) represents the negative control lacking the secondary fluorescent antibody streptavidin-594; (c) myoblasts immediately after biotinylation procedure showing labeling of cell surface proteins; (d) differentiated cultures containing myotubes showing nuclear localisation of proteins that were previously present on the cell surface. B) Isolated nuclei from differentiated cultures probed with streptavidin and LIFR also show nuclear localisation. C) Western immunoblotting for LIFR in cytoplasmic (lanes 1 and 2) and nuclear (lanes 3 and 4) lysates from differentiated cultures. D) Western immunoblotting for histone H3 in cytoplasmic (lanes 1 and 2) and nuclear (lanes 3 and 4) lysates from differentiated cultures.*

## Effect of LIF on survival of donor myoblasts transplanted in mdx muscle

Expression of the gene for the murine homolog of HLA-G, *Qa2*, which is responsible for the ability of LIF to prevent NK cell mediated maternal immune rejection of blastocysts[31] was examined following recombinant LIF treatment in both primary derived and C2C12 myoblasts. Treatment with 10ng/mL recombinant murine LIF, a concentration shown previously to induce anti-apoptotic and anti-myogenic effects on C2C12 and primary murine myoblasts[6] [17], for 24 hours did not significantly alter *Qa2* mRNA levels in either C2C12 or primary myoblasts (Figure 8A). This suggested that LIF may not prevent NK mediated cell death of donor myoblasts in dystrophic muscle through increased *Qa2* expression. However addition of 100ng/mL LIF to the vehicle solution with the male wild-type myoblasts, which were injected into female dystrophic tibialis anterior, increased survival of the donor myoblasts compared to vehicle solution with no LIF (Figure 8B). The survival of male wild-type donor myoblasts was quantitated by qPCR for the presence of the Y chromosome gene *Zfy*. The relative copies of *Zfy* per nanogram of total genomic DNA present in the host muscle immediately after injection was somewhat less than that present in the 200,000 myoblasts prior to injection (Figure 8B), suggesting a slight loss of myoblasts with injection. Relative *Zfy* copies in the host muscle diminished to approximately half 1 day after transplantation indicating rapid loss of donor myoblasts in the dystrophic muscle. Relative *Zfy* copies were further reduced by 3 days after transplantation in the control group without LIF treatment; however the LIF treated group displayed little loss of donor myoblasts since day 1. There were significantly higher *Zfy* copies and therefore surviving donor myoblasts in the dystrophic muscle with LIF treatment compared to control.

**Figure:**
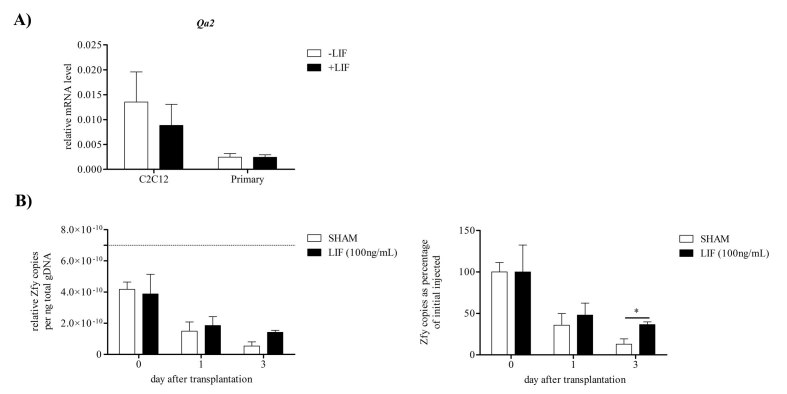
***Figure 8 Effect of LIF on Qa2 mRNA in myoblasts and survival of donor myoblasts in host dystrophic muscle***

A) Transcript levels of the gene Qa2, the murine homolog of HLA-G, in C2C12 and primary myoblasts with or without 10ng/mL LIF treatment for 24 hours. Data was analysed by the pair-wise fixed reallocation randomisation test REST comparing LIF treated to control without LIF treatment. No significant differences were observed, (n=4). B) qPCR detection of the Y chromosome marker Zfy in dystrophic muscle after male myoblast transplantation into the tibialis anterior with or without LIF to determine the effect of 100ng/mL LIF on male donor myoblast survival. Relative Zfy copies normalised to total DNA extracted is presented on the left. Dashed line indicates Zfy copies present in 10µL solution of 200,000 male myoblasts used for injections. Zfy copies per nanogram (ng) of total DNA as a percentage of day 0 Zfy levels are presented on the right. Data was analysed by the pair-wise fixed reallocation randomisation test REST comparing LIF treated to sham without LIF treatment. *P<0.05, (n=6)

## Discussion

These studies present the novel finding that *Lif* mRNA decreases in the quadriceps of wild-type mice in response to two weeks of voluntary exercise. Findings presented herein disagree with previous reports that * Lif* mRNA is up-regulated in mdx muscles compared to wild-type. These studies provide evidence that LIF may promote the survival of myoblasts transplanted into dystrophic muscle. This study also reports the observation that LIFR immunoreactivity localises to newly formed syncytial muscle cells in mdx muscle and the potential for proteins located on myoblast cell membranes, including LIFR, to translocate to nuclei of syncytial muscle cells upon differentiation and fusion.

### *Lif* mRNA expression is decreased in exercised and dystrophic muscles

In wild-type muscle after 2 weeks of exercise transcripts for *Lif* were decreased. This is in contrast to a previous study that reported increased *Lif* mRNA levels after exercise[19], although it is noted that that study was human-based and utilised vastus lateralis muscles sampled soon after a single acute bout of exercise. *Lif* mRNA was up-regulated in this study immediately and 1.5 and 3 hours after the exercise but reduced to control levels by 6 hours, indicating that *Lif* mRNA could be up-regulated within 0-6 hours after exercise. The average 4.5 hours between sampling and last exercise in the present study means that all the exercised mice may have fallen into a time-frame in which *Lif* mRNA is up-regulated soon after exercise. However this was not observed. Therefore, there may be differences in regulation of *Lif* mRNA levels with different exercise regimes, in that transcripts are increased immediately after a single session of exercise, versus down-regulation following multiple and regular exercise bouts over a longer time-frame.

Transcripts for *Lif* and *Lifr* were decreased in sedentary dystrophic muscle, and, unlike wild-type mice, were unaffected by exercise. There are varying reports in the literature about the links of these transcript levels with dystrophic pathology, with some studies indicating an association with pathology in mdx mice[8] and humans[18], and others showing no relationship[38] [39]. Studies demonstrating up-regulation of *Lif* mRNA in mdx muscles by Kurek and colleagues utilised oligonucleotide primer sequences that were only able to detect a single isoform of *Lif* mRNA, whilst the present study utilised primer sequences that could detect both isoforms of *Lif* mRNA and may explain why differences in expression were observed. Functional differences between isoforms are not certain but distribution of the encoded proteins is shown to occur differentially with both secreted extracellularly, one of which is highly soluble whilst the other associates with extracellular matrix[40] [41]. Thus previous studies may have been limited by only detecting a single isoform of *Lif* mRNA whilst the current study provides a broader scope of all potential *Lif* mRNA regulation.

The lack of effect of exercise on *Lif* and *Lifr* mRNA levels in dystrophic muscle suggests that a minimal level of *Lif* mRNA may have been reached. *Lif* mRNA expression has previously been shown to increase following a single damaging event[7] [8] [42] [43] or where muscle is stimulated following a single bout of three hours of concentric exercise[19]. These studies differed somewhat to the present research where repetitive bouts of muscle damage due to the dystrophic phenotype and regular burst of nocturnal exercise interspersed by rest breaks (during the day) led to decreased *Lif* mRNA levels. Therefore the number of damaging or stimulating events most likely causes the change in *Lif* transcript expression where single damage or stimulation events may increase *Lif* expression whilst multiple damage or stimulation events lead to a reduction in *Lif* transcript. Increased LIF protein secretion by cultured myotubes upon electrical stimulation has been suggested to occur rapidly (within 3 hours)[44] and indicates that LIF could be up-regulated rapidly and transiently with muscle stimulation. A recent study published by Srikuea and colleagues also supports rapid and transient up-regulation of *Lif* mRNA with muscle damage[43]. They showed that contusion injury of the gastrocnemius muscle of rats increased *Lif* mRNA for 3-12 hours after injury. A calcium ionophore, ionomycin, by increasing intracellular calcium levels of myotubes in culture can promote both mRNA and protein expression of LIF within six hours[19]. This is similar to mdx mice where repeated damage is caused by increased calcium influx in dystrophic muscle[45]. This could indicate that *Lif* mRNA and protein becomes acutely up-regulated in muscle following a damaging event such as contusion or calcium influx or stimulation by exercise. This appears to occur in a very rapid and transient manner and may have limited detection in experiments over the broader timescales presented herein. Following this, additional and repeated muscle damage or stimulation events may lead to negative feedback on *Lif* mRNA levels. That is, repeated damage or stimulation events may be refractory to increases in *Lif* mRNA and even decrease mRNA below basal levels. This might explain why, despite displaying a trend towards low *Lif* mRNA levels, dystrophic muscle displayed obvious LIF protein immunoreactivity that associated with muscle damage and regeneration.

Although recombinant non-glycosylated LIF protein has been shown to have a relatively short half-life when administered systemically (6-8 minutes in serum), the stability of LIF protein in tissue has not been investigated but it has been speculated that LIF is more likely to act locally and have greater stability in tissue than in circulation[46] [47]. Because endogenously produced LIF is heavily glycosylated and may be produced as an isoform that associates with the extracellular matrix, it may be quite persistent in comparison to recombinant LIF in circulation. Thus LIF protein may persist in skeletal muscle for periods longer than the mRNA which encodes it. Therefore these decreased mRNA levels of *Lif* and *Lifr* in dystrophic and exercised muscle may not necessarily be indicative of LIF and LIFR protein levels present in the muscle.

### LIF and LIFR protein immunolocalisation in normal and dystrophic muscle

Consistent with previous studies examining *Lif* mRNA localisation in normal muscle by *in situ* hybridisation LIF protein immunoreactivity was not apparent in normal muscle from wild-type mice[42]. LIFR protein immunoreactivity however was apparent and appeared to localise to specific areas around myofibre sarcolemma, which was shown to coincide well but not exclusively with the α-bungarotoxin labeled motor endplates of neuromuscular junctions. This suggests that despite minimal to no LIF protein being present in normal muscle, cells of normal muscle tissue may be sensitive to changes in LIF abundance as they express LIFR. Association of LIFR with neuromuscular junctions sits well with the numerous descriptions of the role of LIF in neuronal cell survival, function and signaling[48] [49] [50] [51] [52] [53] [54] [55] [56] [57] [58]. However even with confocal microscopy it was not possible to determine if LIFR was present on the presynaptic or the postsynaptic nerve terminal and therefore difficult to know whether it is motor neurons or myofibres in normal muscle that express the LIFR and are therefore sensitive to LIF. Given the higher frequency of LIFR immunoreactivity compared to the frequency of neuromuscular junctions in a single cross-sectional area it would seem more likely that the LIFR protein immunoreactivity was present on the surface of myofibres.

In the tibialis anterior of mdx mice LIF protein immunoreactivity was not detected at the age of two weeks but was present in numerous mononucleated cells by six weeks of age and had decreased by twelve weeks of age. Previous reports have also suggested in contusion injured muscle that mononucleated cells express *Lif* mRNA[42], supporting the observation that mononucleated cells in damaged muscle tissue may produce and secrete LIF protein. The high number of mononucleated cells positive for LIF immunoreactivity at six weeks of age coincided well with increased myogenic and inflammatory transcripts*. * Whilst at two weeks of age, when no histopathology could be observed and there was no up-regulation of markers that associated with pathology, LIF protein immunoreactivity could not be detected. Therefore the presence of LIF protein does appear to associate with the onset of pathology in mdx mice and these results do support a role for LIF in regenerating and diseased muscle.

### LIFR protein immunoreactivity localises to nuclei of newly formed syncytial muscle cells

When examining LIFR protein immunoreactivity in the mdx tibialis anterior it was observed that the immunoreactivity appeared to co-localise with the central nuclei of regenerated syncytial muscle cells. Given the odd nature of a cell surface receptor apparently localising to nuclei this was investigated both *in vitro* and in another model where syncytial muscle cells are formed by injury and regeneration from notexin. In all cases LIFR protein immunoreactivity localised to the nuclei of syncytial muscle cells and appeared most obvious in syncytial muscle cells showing more intense cytoplasmic desmin immunoreactivity indicating that the syncytial cells were formed more recently from the fusion of myogenic precursors. Thus it seemed that the LIFR which is normally present on the cell surface of myoblasts appaeared to translocate to the nuclei upon fusion into syncytial myotubes. Experiments biotinylating cell surface proteins on myoblasts that subsequently translocated to the nuclei in fused myotubes provided evidence that this is a possible path that cell surface proteins from myoblasts may take. Theoretically this biotinylation could have labeled any proteins present on the cell surface of myoblasts that have a primary amine exposed extracellularly. Therefore this finding could represent cell surface receptors, structural proteins, ion channels and numerous other protein types that are translocating from the cell surface to nuclei with myogenic differentiation. We are unaware of any other studies that have reported differentiation/fusion-associated nuclear translocations of cell surface proteins in muscle cells and further studies on identifying the nature of these proteins may be important to further understanding the regulation of the myogenic process.

This is not the first description of cell surface receptors translocating to nuclei within cells. The receptors for epidermal growth factor (EGF) and fibroblast growth factors (FGFs) can translocate from the cell surface to nuclei within cells in a ligand dependent manner[59] [60] [61] [62]. There are also other reports that LIFR may localise to nuclei within other cell and tissue types[55] [63] [64]. Some of these used antibodies that were clearly defined in the methodology and were different from the one used herein. It is therefore unlikely that all of these observations, including that presented herein, were due to a protein not related to LIFR with immunoreactivity to all the LIFR antibodies used. It was also interesting that this occurred in another type of syncytial cell, osteoclast-like cells, which are formed by the fusion of monocyte cells. Evidence provided here suggests that LIFR nuclear translocation occurs due to myoblast differentiation or fusion. Therefore nuclear translocation of the LIFR may represent a mechanism by which cells can regulate LIF activity during events such as cell differentiation or fusion. The potential purpose or function of nuclear translocation of the LIFR however is uncertain; whether translocation of LIFR still allows kinase activity or simply prevents extracellular LIF from binding to LIFR on the cell surface is unknown.

Despite the highly reproducible LIFR nuclear localisation in myotubes, as detected by immunofluorescence, this was not corroborated by Western blotting of nuclear fractions. In previous reports examining nuclear translocation of EGF and FGF receptors typically full-length receptor protein was observed within nuclei. In the present report it was observed that immunoreactive bands were present within the nuclei of myotubes, but none of these corresponded to full-length 190kDa LIFR. This raises the possibility that post-translational modifications of the LIFR may be necessary for nuclear translocation. Hanson and colleagues have reported nuclear localisation of LIFR and also found several immunoreactive bands that could correspond to shorter isoforms of the LIFR protein of approximately 50, 80 and 90kDa[64] supporting findings herein. Endocytosis of the LIFR may also be necessary to explain nuclear translocation of the LIFR. Endocytosis of the LIFR complex has been suggested as necessary to explain retrograde signaling of STAT3 by LIF from distal neurites to cell bodies in culture[58]. Thus nuclear translocation of LIFR may require post-translational modifications and endocytosis in order to occur. There is a close physical association between some cellular organelles, such as the golgi and endoplasmic reticulum, and nuclei. It is therefore possible even using methods described herein with separated intact nuclei, that peri-nuclear localisation may not be distinguishable from nuclear localisation. Thus the possibility of peri-nuclear localisation of LIFR cannot be discounted. However the technically simple and easily reproducible translocation of LIFR with differentiation of C2C12 myoblasts would allow for further examination of this phenomenon.

### LIF promotes survival of donor myoblasts in dystrophic muscle

Addition of exogenous LIF to donor myoblasts partially protected them between 1 and 3 days following transplantation into dystrophic host muscle. Recombinant LIF treatment did not promote up-regulation of *Qa2* mRNA, which is up-regulated *in utero* by LIF and responsible for preventing NK mediated immune rejection of blastocysts[29] [30] [31] in myoblasts. This suggested that LIF was not likely to promote survival of donor myoblasts through up-regulation of *Qa2* and subsequent inhibition of NK cell mediated death. Depletion of host NK cells in dystrophic mice has been shown to promote survival of donor myoblasts, but only at seven days after transplantation and not earlier[28]. Depletion of host neutrophils however promoted survival of donor myoblasts three days after transplantation, the same effect as LIF. Knockout of the *Lif* gene has been shown to increase neutrophil numbers present in inflamed dermis[65] suggesting LIF may modulate inflammatory neutrophil numbers. It has also been observed that inhibition of LIF during notexin induced regeneration of the tibialis anterior increases neutrophil numbers (author’s own unpublished observations). Therefore LIF may be able to reduce host neutrophil numbers and this might explain how LIF promoted survival of donor wild-type myoblasts in dystrophic host muscle and promoted dystrophin expression[25] [26].

Transplantation of myogenic cells into dystrophic muscle as a potential therapy has progressed beyond the use of only myoblasts due to disadvantages associated with the use of myoblasts and advantages present with use of other cell types such as skeletal muscle, blood and bone derived stem cells[66]. One key factor however that is required for all potential donor cells is that they can survive long enough to achieve the goal of differentiating and fusing into host muscle so dystrophin can be expressed. Data presented herein suggested that LIF may promote survival of donor cells by manipulating the host environment. Previous studies that suggested LIF provided the greatest benefit on dystrophin expression following myoblast transplantation occurred when LIF was being released into the host muscle by alginate gels[25] and supports LIF affecting the environment of the host muscle. Therefore, as an adjunct to transplantation, LIF could provide benefit enhancing the survival of any donor cells used for transplantation by modulating inflammation in the host dystrophic muscle.

### Conclusions

These studies demonstrated that although regulation of LIF protein coincides with pathology in the mdx mice and supports a role for LIF in regeneration and disease of skeletal muscle, *Lif* mRNA levels are not particularly indicative of protein abundance. Decreases in *Lif* mRNA with exercise or in dystrophic muscle may however suggest that a change in protein level has occurred, though it is not directly proportional to the change in mRNA. These studies also present the novel observation that LIFR protein may translocate from the cell surface of myoblasts to nuclei of syncytial muscle cells upon fusion, which could have potential implications for the regulation of LIF activity. LIF also appears to promote survival of transplanted myoblasts in dystrophic muscle in a manner that suggests modulation of neutrophils. Thus LIF may have an important role in inflammation of skeletal muscle.

## Acknowledgments

The authors acknowledge the contributions of Kitipong Uaesoontrachoon and Charles Pagel for providing oligonucleotide primer sequences for the genes * Myod1* and *Myog*. Su Toulson should also be acknowledged for her contributions toward the sampling of mdx mice and subsequent processing of those samples.

## Funding information

This work was funded by the Muscular Dystrophy Association of Australia (MDA).

## Competing interests

The authors have declared that no competing interests exist.
